# A Static Magnetic Field Inhibits the Migration and Telomerase Function of Mouse Breast Cancer Cells

**DOI:** 10.1155/2020/7472618

**Published:** 2020-05-11

**Authors:** Zhu Fan, Pingdong Hu, Lekang Xiang, Ying Liu, Rongqiao He, Tao Lu

**Affiliations:** ^1^School of Life Sciences, Beijing University of Chinese Medicine, Beijing 100029, China; ^2^State Key Laboratory of Brain and Cognitive Science, Institute of Biophysics, CAS, Beijing 100101, China; ^3^University of Chinese Academy of Sciences, Beijing 100101, China

## Abstract

Static magnetic field (SMF) has a potential as a cancer therapeutic modality due to its specific inhibitory effects on the proliferation of multiple cancer cells. However, the underlying mechanism remains unclear, and just a few studies have examined the effects of SMF on metastasis, an important concern in cancer treatment. In this study, we evaluated the effects of moderate SMF (~150 mT) on the proliferation and migration of 4T1 breast cancer cells. Our results showed that SMF treatment accelerated cell proliferation but inhibited cell migration. Further, SMF treatment shortened the telomere length, decreased telomerase activity, and inhibited the expression of the cancer-specific marker telomerase reverse transcriptase (TERT), which may be related to expression upregulation of e2f1, a transcription repressor of TERT and positive regulator of the mitotic cell cycle. Our results revealed that SMF repressed both, cell migration and telomerase function. The telomerase network is responsive to SMF and may be involved in SMF-mediated cancer-specific effects; moreover, it may function as a therapeutic target in magnetic therapy of cancers.

## 1. Introduction

Static magnetic fields, such as the natural geomagnetic field (GMF, ~50 *μ*T) and artificial magnetic fields produced by magnetic materials or instruments, are widely present in the environment. Magnetic fields of different intensities play an important role in the diagnosis and treatment of diseases [1]. For example, strong magnetic field (>1 T) are used in magnetic resonance imaging to help diagnose diseases, whereas moderate magnetic fields (1 mT–1 T) are widely used in the alternative and complementary treatment of various diseases [2–4]. Potential applications of SMF in cancer treatments have been indicated because of the specific inhibitory effects of SMF on the growth of multiple types of cancer cells. However, there is no consensus regarding the effect of SMF on cancer cells, and understanding the effects and underlying mechanism of SMF is critical before this method can be clinically applied.

Many studies have shown that SMF inhibits the proliferation of multiple tumor cells, and tumor cells could be more sensitive to magnetic fields compared to nontumor cells. Zhang et al. [5] treated seven human solid cancer cell lines and five human noncancer cell lines with 1-T magnetic field and found that the SMF significantly affected the proliferation of cancer cells but not noncancer cells. These results indicate that cancer-specific molecules are involved in cell proliferation regulation by SMF. Telomerase is cancer-specific marker rarely expressed in noncancer cells, and telomerase activation is a key factor in maintaining the telomere length for the immortal division of cancer cells [6]. Therapies targeting telomeres trigger DNA damage responses in tumor cells and lead to aging or apoptosis [7–9]. We speculate that SMF may cause alterations in telomerase to affect the proliferation of cancer cells.

In addition, metastatic cancer is more fatal than nonmetastatic cancer [10]. However, few studies have evaluated the effects of moderate SMF on the migration of cancer cells. We previously found that GMF shielding (<200 nT) accelerated the proliferation but inhibited the cell motility of human neuroblastoma cells, but the specific effects were difficult to determine in the absence of a molecular marker [11, 12]. In addition to its classic role in affecting telomere length, telomerase is also related to the migration of cancer cells, and the expression of the subunit telomerase reverse transcriptase (TERT) can be used to distinguish benign from malignant tumors. The overexpression of TERT promotes cell migration, whereas a reduction in TERT expression results in decreased cell migration and adhesion [13, 14]. Coanalysis of the effects of SMF on cell proliferation, migration, and telomeres will increase the understanding of the effects and underlying mechanisms, as well as the risk of magnetic therapy.

Breast cancer cells are commonly used as a model for analyzing cancer metastasis and are sensitive to SMF treatment. SMF can inhibit the proliferation of different breast cancer cells and enhance the efficacy of specific chemotherapy drugs both *in vivo* [15] and *in vitro* [16–18]. Therefore, in this study, we evaluated the effects of a moderate SMF (~150 mT) on 4T1 breast cancer cells. We found that SMF treatment accelerated cell proliferation but inhibited cell migration and telomerase function, which were related to decreased telomerase activity and TERT expression. Our findings revealed that cancerous features of cells were reduced by SMF. The telomerase network responds to SMF and may act as a target in magnetic therapy for breast cancer.

## 2. Materials and Methods

### 2.1. Cell Culture and Treatment

Mouse breast cancer cell line 4T1 was purchased from the Cell Culture Bank of the Chinese Academy of Sciences' Culture Collection Committee. Cells were maintained in Dulbecco's Modified Eagle Medium (DMEM) (high d-glucose) supplemented with 10% fetal bovine serum (FBS; Gibco, Grand Island, NY, USA), 100 U/mL penicillin, and 100 *μ*g/mL streptomycin (Gibco) and cultured at 37°C with 5% CO_2_. The medium was changed every 2 days.

For magnetic field treatment, cells in the logarithmic growth phase were seeded at a density of 1 ×10^4^ cells/mL at 1 mL/well in a 12-well plate except for in the Transwell assay. After incubation for 12 h, the cells were exposed to a moderate SMF. Cells cultured in the GMF area without SMF treatment were used as controls.

### 2.2. MF Conditions

**Figure 1:**
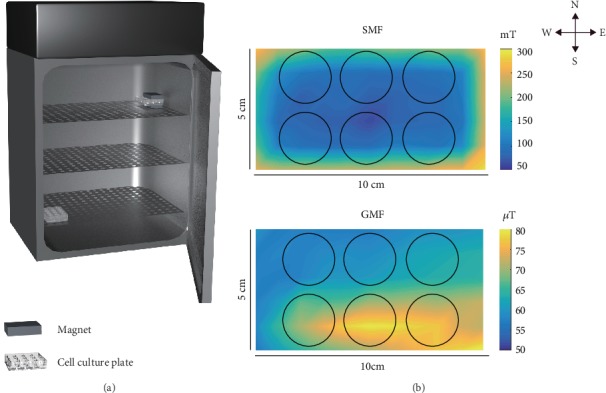
Experimental setups for SMF treatment. (a) The cell incubation system for MF treatment. A pair of magnets placed upon and under the cell plate provided the SMF. GMF control and SMF-exposed cells were incubated on different floors. (b) Distributions of the magnetic fields (vector sum) at the bottom plane of the cell plate for SMF (~153 mT) and GMF (~65 *μ*T) treatment. Magnetic field was measured at an interval of 1 × 1 cm. (Black circles represent the exposed cell wells of a 12-well plate).

The SMF and GMF conditions were set up in a CO_2_ incubator (width × height × depth: 63 × 92 × 69 cm, INCO 153 med, Memmert, Schwabach, Germany) on different layers. The untreated GMF control samples were placed at a position with an average SMF of 65.08 ± 7.18 *μ*T, which is similar to the local magnetic field in the laboratory. A 150-mT rectangular magnet (neodymium iron boron, 10 × 5 cm, Genchang, Jiangsu, China) was applied for SMF treatment. The cell plate was placed between a pair of magnet blocks as shown in Figure 1(a), and no more than 2 sets of SMF/GMF plates were placed in one incubator to prevent the MFs from disturbing the other plates. The average SMF was 153.9 ± 72.0 mT (vector sum, Table 1), which was calculated from measurements performed at an interval of 1 × 1 cm on the bottom of the plate attached to the magnet (Figure 1 and Table 1). The five positions represent the mean field strength in the cell culture wells in the southeast, southwest, northeast, and northwest, and center of the magnetic field. The SMFs were measured with a permanent magnet digital gauss meter (HT20, Shanghai Hengtong, Shanghai, China).

### 2.3. Cell Proliferation Assays

Cell proliferation was analyzed hemocytometry for cell counting and in a cell division assay by carboxyfluorescein diacetate succinimidyl ester (CFSE) staining.

CFSE staining was conducted according to the manufacturer's instructions (Cat. No. 565082, BD Horizon, BD Biosciences, Franklin Lakes, NJ, USA). Briefly, the cells were stained with 25 *μ*M CFSE for 20 min at 37°C. After two washes with phosphate-buffered saline, the CFSE-stained cells were seeded into 12-well plates for magnetic field treatment as described in section 2.1. The cells were collected after 24 and 48 h of exposure, and CFSE fluorescence was measured with a FACS Caliburflow cytometer (BD Biosciences) and analyzed with the Cell Quest Pro software.

### 2.4. Wound Healing Assay

Cells were seeded into 12-well plates containing DMEM with 10% FBS and grown into monolayers. After confluence reached greater than 90%, wounds were made with a pipette tip to form a cross area on the cells. Detached cells were removed using serum-free DMEM, and 4 T1 cells were exposed to an SMF for 24 h. The wound width was imaged at 0 (D0) and 24 h (d) and analyzed using the ImageJ software (NIH, Bethesda, MD, USA). The migration efficiency was calculated as (*D*0–*d*)/*d* × 100%.

### 2.5. Transwell Assay

Cell migration was detected in 24-well Transwell chambers (Corning, Inc., Corning, NY, USA). 4T1 cells (5 × 10^4^ cells) were resuspended in DMEM (200 *μ*L) with 1% FBS added to the upper chamber, and 400 *μ*L DMEM with 10% fetal bovine serum added to the lower chamber. 4T1 cells were exposed to GMF and SMF for 24 h. After fixation, the cells were stained with 0.1% Hoechst and photographed with a DM5000 B microscope (Leica, Wetzlar, Germany). Five randomly selected fields of each membrane were counted. Cell numbers were calculated using the ImageJ software.

### 2.6. Reverse Transcription Real-Time Quantitative Polymerase Chain Reaction (RT-qPCR)

The expression of *TERT*, *e2f1*, *mzf1*, and *sp1* was analyzed by RT-qPCR. After 72 h of exposure, RNA was extracted using a RNeasy Mini kit according to the manufacturer's instructions (Qiagen, Hilden, Germany). Reverse transcription from total RNA was performed to synthesize cDNA (Qiagen), and a Rotor gene Q PCR Cycler (Qiagen, Valencia, CA, USA) was used for detection. Primer sequences were designed using Primer bank (https://pga.mgh.harvard.edu/primerbank/) [19], as shown in Table 2. *Gapdh* was used as an internal control.

### 2.7. Telomerase Activity Assay

The telomerase activity of the cell extracts was measured with a TRAPeze RT Telomerase Detection Kit (Cat. No. S7710; Millipore, Billerica, MA, USA). The cells were inoculated into 12-well plates, and the inoculation density and treatment conditions were the same as those described in section 2.1. After 72 h of treatment, we tested the telomerase activity according to the manufacturer's instructions. Each assay mixture consisted of 5 *μ*L 5x TRAPeze RT reaction mixture, 17.6 *μ*L PCR grade water, 0.4 *μ*L 50x TITANIUM Taq DNA polymerase (Clontech, Mountain View, CA, USA), and 2 *μ*L cell extract or control template. A series of diluted TSR8 control templates was prepared in CHAPS lysis buffer to prepare a standard curves. Two additional replicate wells were used for each sample. The PCR amplification of the telomerase substrate was detected by real-time PCR with a Rotor gene Q PCR Cycler (Qiagen, Valencia, CA, USA) using the following cycle parameters: 30 min at 30°C, 2 min at 95°C, 45 cycles of 94°C for 15 s, 59°C for 1 min, and 45°C for 30 s. The linear plot of the log10 and *Ct* values from the amount of the TSR8 control template standard was used to determine the amount of expanded telomerase substrate produced in each well from the telomerase activity of 2 *μ*L cell extract within 30 min. The average of the two replicate wells for each sample was calculated. This number was divided by the amount of protein (mg) contained in the 2 *μ*L extract and then divided by 30 min to determine the amount of extended telomerase substrate produced in every minute per milligram protein.

### 2.8. Telomere Length Detection

After 72 h of treatment, the cells were collected, and DNA was extracted using a DNeasy Blood and Tissue Kit (Cat. No. 69504, Qiagen). The average telomere length of total genomic DNA was determined by qPCR as described by Cawthon [20] and Callicott et al. [21] The telomere primer sequences (5′-3′) were as follows: forward, CGGTTTTTTGGTTTTGGTTGGTTGGTTGGTTGGGTGTGTGTGTGT; and reverse, GGGTTGGCCTTACHCCTTACHCCTTACHCCTTACHCCTTACHCCTTACHCT. The reference control gene primer (mouse 36B4 single-copy gene) sequences were as follows: forward, TGAAGTGCTTGACATCACGAGGA; and reverse, CTGCAGACATCGCTGGCAAATT. An equal amount of DNA (35 ng) was used for each reaction and both, the telomere and 36B4 gene, were amplified under the same conditions. For each PCR, a standard curve was generated by serially diluting a known amount of DNA. The telomere (T) signal was normalized to the signal obtained from a single-copy (S) gene to generate a T/S ratio indicating the relative telomere length.

### 2.9. Bioinformatics

Transcription Factor Database TRRUST (version 2) [22] (https://www.grnpedia.org/trrust/) is a manually curated database of transcription factors (TF) and TF-target regulatory relationships, which contains 8,444 and 6,552 TF-target regulatory relationships of 800 human TFs and 828 mouse TFs. We input the transcription factors of interest into TRRUST (version 2), selected “mouse” as the species, obtained all genes regulated by the TFs, and classified the genes regulated according to “Activation,” “Repression,” and “Unknown”.

Metascape [23] (http://www.metascape.org/, 2019/8/14) was used to further analyze the gene function and enrichment pathway. We uploaded the gene list to the website, selected “M. musculus” for “Input as fields” and “Analysis as fields”, and analyzed the gene according to “Custom Analysis.” In Custom Analysis, we only checked “GO Biological Processes” in the “Pathway” option of “Membership” and “Enrichment”; the other options were used with default values, Min overlap: 3, P value cutoff: 0.01, Min enrichment: 1.5.

### 2.10. Statistical Analysis

Each experiment was repeated at least three times in triplicate. Unless otherwise indicated, *t* test was used to compare the means. Results showing *P* values of less than 0.05 were considered as significant.

## 3. Result

### 3.1. SMF Treatment Accelerated Proliferation and Inhibited Migration of 4 T1 Cells

**Figure 2:**
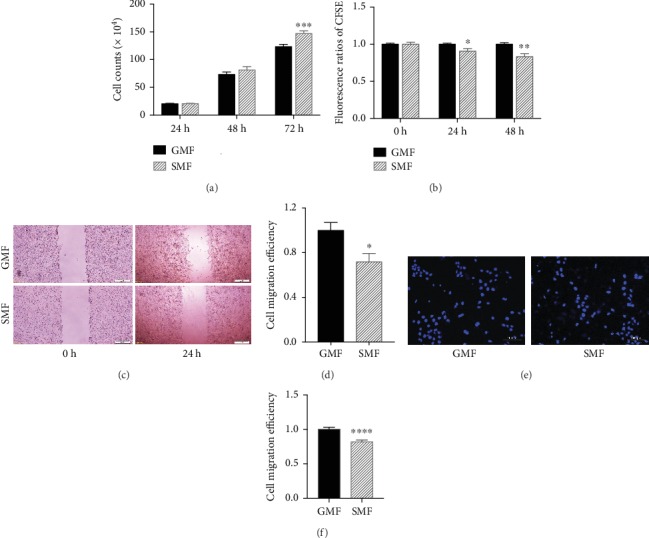
SMF treatment accelerated proliferation and inhibited migration of 4T1 cells. (a) Cell numbers counted following SMF exposure to different magnetic fields for 24, 48, and 72 h (h). (b) The proliferation rates of 4T1 cells shown by CFSE fluorescence ratio of SMF/GMF at 24 and 48 h of exposure. (c) Representative images of the wound width and (d) migration efficiency of the SMF and GMF cell at 0 and 24 h of the exposure in the wound healing assay. Wound healing assays and Transwell assays (e) were used to detect the migration ability of cells. (e) Representative fluorescent images showing the nuclei (blue, stained by Hoechst) of the migrated cells exposed in GMF and SMF for 24 h. (f) Compared to the GMF group, SMF treatment significantly inhibited cell migration. Data are the means ± sem from three independent experiments (*n* = 3). ∗*P* < 0.05; ∗∗*P* < 0.01; ∗∗∗∗*P* < 0.0001, compared to the GMF group. SMF: static magnetic field; GMF: geomagnetic field.

The effect of SMF treatment on the proliferation of 4T1 cells was analyzed by cell counting and CFSE staining (Figures 2(a) and 2(b)). First, we monitored the number of 4T1 cells exposed to the magnetic field for 24, 48, and 72 h. The results showed that the cell number in the SMF group was the same as that in the GMF group at 24 h, and higher at 48 h (11.02%), reaching a significant increase at 72 h (19.28%) of treatment. These effects on proliferation acceleration were confirmed by CFSE staining, with the rate of cell division inversely proportioned to the fluorescence intensity remaining in the daughter cells (Figure 2(b)). The fluorescence ratio in SMF-treated cells was significantly lower than that in the GMF group at 24 h of exposure (10.39%), and the reduction became greater at 48 h (20.16%). Thus, the proliferation of 4T1 cells was accelerated by SMF, and the cell response to MF was detectable within 24 h.

The effects of SMF treatment on the migration of 4T1 cells were measured in wound healing and Transwell assays at 24 h of exposure in serum-free and low-serum medium, respectively, to abolish the effect on proliferation. As observed in the wound healing assays (Figures 2(c) and 2(d)), the width of the “wound” healed in SMF was smaller than that in the GMF control, and the cell migration efficiency in SMF was only 71.68% of that in the GMF (*P* < 0.05). The results of the Transwell assays (Figures 2(e) and 2(f)) also revealed fewer transported cells in the SMF than in the GMF group (*P* < 0.0001), confirming that SMF treatment inhibited the migration ability of 4T1 cells.

### 3.2. SMF Treatment Decreased Telomerase Function in 4 T1 Cells

Considering the accelerated division of 4 T1 cells in the SMF, it is important to evaluate the effect on immortality, a characteristic of cancer cells. Telomerase is rarely expressed in most normal cells but is activated in more than 90% of tumor cells [24, 25] and is a key factor in maintaining the proliferative ability and telomere length of tumor cells and determining cell life. Thus, we next evaluated the telomerase activity, telomere length, and expression of telomere-associated proteins at 72 h of MF exposure, when the greatest effect on cell proliferation was detected.

**Figure 3:**
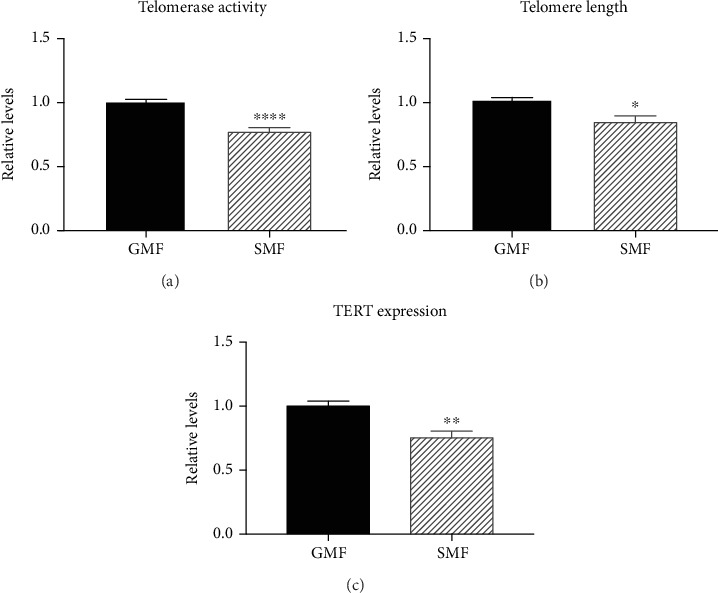
SMF treatment decreased telomerase function in 4T1 cells after 72 h exposure. (a) Relative telomerase activity, (b) relative telomere length, and (c) relative mRNA expression of TERT in GMF- or SMF-treated cells; data are the means ± sem normalized to the GMF control (*n* = 9 from three independent experiments). ∗*P* < 0.05, ∗∗*P* < 0.01, and ∗∗∗∗*P* < 0.0001 compared to the GMF group.

Our results showed that SMF treatment significantly inhibited telomerase activity and shortened telomeres in 4T1 cells compared to in the GMF group (Figures 3(a) and 3(b)), indicating decreased division related to telomerase and a tendency for accelerated exit from limitless cancerous growth.

Moreover, compared to the GMF group, the expression of telomerase (telomerase reverse transcriptase, *TERT*) was downregulated, as demonstrated in the RT-qPCR assay (Figure 3(c)). These data indicate that decreased telomerase activity following SMF involves the response of upstream expression regulators rather than effects on telomerase alone.

### 3.3. SMF Treatment Upregulates e2f1 Expression in 4 T1 Cells

**Figure 4:**
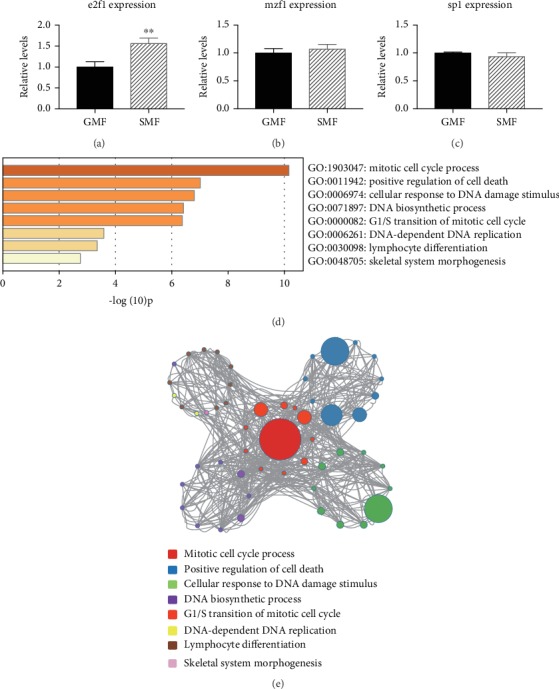
SMF treatment upregulates e2f1 expression in 4T1 cells. (a–c) The relative expression of transcription factors e2f1, mzf1, and sp1 in SMF-treated cells at 72 h of exposure detected by RT-qPCR. Data are the means ± sem normalized to GMF control, *n* = 9 from 3 independent experiments, ∗∗*P* < 0.01. (d) Columns illustration shows target genes activated by e2f1 from GO enrichment analysis, and the terms with *P* < 0.01 are listed. (e) Network relationship between enriched terms. The circle of the same color represents the same term; the size of the circle is positively correlated with the number of genes enriched in this term.

To further explore the SMF-responsive regulator of *TERT*, we examined the expression levels of TFs upstream of *TERT*, such as the activating TF sp1 and the inhibitory TFs e2f1 [26] and mzf1, by RT-qPCR. As shown in Figures 4(a)–4(c), the expression level of e2f1 was significantly higher in SMF-treated cells than in the GMF control, whereas the other TFs did not change significantly.

To determine whether e2f1 mediates the response of 4 T1 cells to the magnetic field, GO enrichment analysis was performed on genes activated by e2f1 (Figure 4(d)). The terms sorted based on the *P* values showed that 4 of the top 6 terms were related to the cell cycle, and the top was related to the mitotic cell cycle process, which may partially explain the accelerated proliferation of tumor cells. To further examine the relationship between terms, we chose a subset of enriched terms and constructed a network graph (Figure 4(e)). We found that the top three biological processes were enriched in mitotic cell cycle process, positive regulation of cell death, and cellular response to DNA stimulus. The possible activation of cell death indicates a tendency for the fate change of immortalized cancer cells.

## 4. Discussion

In this study, we examined the potential of SMF in cancer treatment by coanalysis of the effect on proliferation, migration, and telomeres and revealed the role of telomerase in response to SMF. We found that a moderate SMF (~150 mT) accelerated cell proliferation but inhibited breast cancer cell migration and shortened telomere length, which was associated with decreased telomerase activity and expression of TERT, as well as corresponding upregulation of e2f1 expression.

This is the first study to demonstrate an association of telomerase and the effects on cell proliferation and migration under SMF treatment. E2f1 is a transcription repressor of TERT and positive regulator of the mitotic cell cycle, as shown by GO enrichment analysis. Its upregulation may lead to downregulation of TERT and the acceleration of proliferation. Decreased TERT can mediate migration repression and telomere shortening. Thus, SMF treatment may antagonize tumor growth by restricting the uncontrolled division in addition to inhibit cell proliferation and cause cell death.

In this study, the proliferation and division of 4T1 cells were accelerated by SMF treatment, which contrasts previously reported results [27, 28]. This was expected, as the exact effects of SMFs on cells are largely dependent on the cell types and magnetic conditions [5]. Although SMF shows anticancer potential because of its ability to specifically inhibit the proliferation of cancer cells, accelerated proliferation may improve the efficacy of some chemotherapy drugs against rapidly dividing cells.

The inhibition effect on 4T1 migration was consistent with that observed in our previous study, as well as a few others evaluating different cell types and treatment condition [29, 30], suggesting the potential of using a SMF to inhibit metastasis in cancer treatment.

A unique feature of tumor cells is immortalization, in which telomerase activation is a key factor. Active telomerase, a reverse transcriptase, can directly increase telomere length [6]. As SMF can accelerate cell proliferation and reduce telomerase activity, further studies are needed to determine whether prolonging the SMF can shorten the telomere to a critical length and stop division, thus leading to the aging of tumor cells. Previous studies showed that SMF can accelerate senescence and shorten lifespan in nematodes. For example, Hung et al. [31] found that after 200 mT of steady SMF treatment, the development rate of wild-type nematodes was increased by 20–31%, and the average lifespan decreased from 31 to 24 days. After SMF treatment, pathways involving development and senescence-related genes, such as *let-7*, *clk-1*, *unc-3*, and *age-1*, were significantly upregulated in nematodes [32]. Nematodes exposed to 8.5 T SMF also showed significant acceleration of aging and increased expression of superoxide dismutase-3.

Overall, SMF treatment inhibits cell migration and may accelerate/induce the exit from immortalizing division by repressing telomerase activity in tumor cells. The telomerase network can respond to the SMF and may be involved in cancer-specific effects and function as a target in magnetic therapy.

## Figures and Tables

**Table 1:** Magnetic field conditions^a^.

Group	Position	∣*B*∣^b^	∣*B*x∣^c^	∣*B*y∣^d^	∣*B*z∣^e^
SMF (mT)	Center	75.92 ± 7.76	6.5 ± 3.14	2.83 ± 1.46	75.5 ± 7.91
	Southeast	97.97 ± 13.16	24.83 ± 6.06	15.66 ± 11.17	92.83 ± 14.45
	Southwest	88.96 ± 10.82	4.5 ± 2.75	17.66 ± 16.42	85.66 ± 9.14
	Northeast	95.5 ± 10.39	14.83 ± 6.71	4.16 ± 4.41	94 ± 9.52
	Northwest	97.55 ± 16.2	3.5 ± 2.98	6.33 ± 4.71	97.16 ± 15.56
	Average	153.9 ± 72.0			

GMF (*μ*T)	Center	64.85 ± 4.27	25.86 ± 3.96	56.93 ± 5.45	16.28 ± 2.07
	Southeast	92.62 ± 0.63	23.45 ± 2.3	55.65 ± 0.43	17.63 ± 0.19
	Southwest	73.42 ± 3.11	29.71 ± 4.9	63.11 ± 6.63	21.13 ± 4.37
	Northeast	58.29 ± 0.88	20.46 ± 1.83	51.09 ± 1.27	19.05 ± 0.85
	Northwest	67.4 ± 4.46	18.93 ± 14.05	60.6 ± 10.76	14.51 ± 2.79
	Average	65.08 ± 7.18			

^a^ Data are the mean ± sd of measurement reads at the same layer; ^b^ Net static magnetic field (vector sum of the three directions); ^c–e^ Magnetic field directions: *x*, south to north; *y*, east to west; *z*, downward. ^∗^: $|B|=\sqrt{{\left(Bx\right)}^{2}+{\left(By\right)}^{2}+{\left(Bz\right)}^{2.}}$

**Table 2:** Primer sequences used for RT-qPCR.

Target gene	Forward primer (5′-3′)	Reverse primer (3′-5′)
*TERT*	GCACTTTGGTTGCCCAATG	GCACGTTTCTCTCGTTGCG
*E2f1*	CTCGACTCCTCGCAGATCG	GATCCAGCCTCCGTTTCACC
*Mzf1*	AATTGCCACTGAACCTACCAATG	TGTCGCTATGAGGAGAGGTCT
*Sp1*	GCCGCCTTTTCTCAGACTC	TTGGGTGACTCAATTCTGCTG
*Gapdh*	AGGTCGGTGTGAACGGATTTG	TGTAGACCATGTAGTTGAGGTCA

## Data Availability

All data included in this study are available upon request from the corresponding author.

## References

[1] Ghodbane S., Lahbib A., Sakly M., Abdelmelek H. (2013). Bioeffects of Static Magnetic Fields: Oxidative Stress, Genotoxic Effects, and Cancer Studies. *BioMed Research International*.

[2] Alfano A. P., Taylor A. G., Foresman P. A. (2001). Static magnetic fields for treatment of fibromyalgia: a randomized controlled trial. *Journal of Alternative and Complementary Medicine*.

[3] Eccles N. K. (2005). A critical review of randomized controlled trials of static magnets for pain relief. *Journal of Alternative and Complementary Medicine*.

[4] Salvatore J. R., Harrington J., Kummet T. (2003). Phase I clinical study of a static magnetic field combined with anti-neoplastic chemotherapy in the treatment of human malignancy: initial safety and toxicity data. *Bioelectromagnetics*.

[5] Zhang L., Ji X. M., Yang X. X., Zhang X. (2017). Cell type- and density-dependent effect of 1 T static magnetic field on cell proliferation. *Oncotarget*.

[6] Lee H. W., Blasco M. A., Gottlieb G. J., Horner J. W., Greider C. W., DePinho R. (1998). Essential role of mouse telomerase in highly proliferative organs. *Nature*.

[7] Li G., Shen J., Cao J. (2018). Alternative splicing of human telomerase reverse transcriptase in gliomas and its modulation mediated by CX-5461. *Journal of Experimental & Clinical Cancer Research*.

[8] Zheng X. H., Nie X., Fang Y. (2017). A Cisplatin Derivative Tetra-Pt(bpy) as an Oncotherapeutic Agent for Targeting ALT Cancer. *Journal of the National Cancer Institute*.

[9] Rankin A. M., Faller D. V., Spanjaard R. A. (2008). Telomerase inhibitors and T-oligo' as cancer therapeutics: contrasting molecular mechanisms of cytotoxicity. *Anti-Cancer Drugs*.

[10] Bohl C. R., Harihar S., Denning W. L., Sharma R., Welch D. R. (2014). Metastasis suppressors in breast cancers: mechanistic insights and clinical potential. *Journal of Molecular Medicine*.

[11] Mo W.-C., Zhang Z.-J., Wang D.-L., Liu Y., Bartlett P. F., He R. Q. (2016). Shielding of the geomagnetic field alters actin assembly and inhibits cell motility in human neuroblastoma cells. *Scientific Reports*.

[12] Mo W., Liu Y., Bartlett P. F., He R. (2014). Transcriptome profile of human neuroblastoma cells in the hypomagnetic field. *Science China Life Sciences.*.

[13] Liu H., Liu Q., Ge Y., Zhao Q., Zheng X., Zhao Y. (2016). hTERT promotes cell adhesion and migration independent of telomerase activity. *Scientific Reports*.

[14] Maggisano V., Celano M., Lombardo G. E. (2017). Silencing of hTERT blocks growth and migration of anaplastic thyroid cancer cells. *Molecular and Cellular Endocrinology*.

[15] Gray J. R., Frith C. H., Parker J. D. (2000). In vivo enhancement of chemotherapy with static electric or magnetic fields. *Bioelectromagnetics*.

[16] Lin T., Wan L., Qi X., Shi W., Lin J. (2014). A moderate static magnetic field enhances TRAIL-induced apoptosis by the inhibition of Cdc2 and subsequent downregulation of survivin in human breast carcinoma cells. *Bioelectromagnetics*.

[17] Aljarrah K., Mhaidat N. M., al-Akhras M. A. H. (2012). Magnetic nanoparticles sensitize MCF-7 breast cancer cells to doxorubicin-induced apoptosis. *World Journal of Surgical Oncology*.

[18] Luo Y., Ji X., Liu J. (2016). Moderate intensity static magnetic fields affect mitotic spindles and increase the antitumor efficacy of 5-FU and Taxol. *Bioelectrochemistry*.

[19] Wang X., Spandidos A., Wang H., Seed B. (2012). PrimerBank: a PCR primer database for quantitative gene expression analysis, 2012 update. *Nucleic Acids Research*.

[20] Cawthon R. M. (2002). Telomere measurement by quantitative PCR. *Nucleic Acids Research*.

[21] Callicott R. J., Womack J. E. (2006). Real-time PCR assay for measurement of mouse telomeres. *Comparative Medicine*.

[22] Han H., Cho J. W., Lee S. (2018). TRRUST v2: an expanded reference database of human and mouse transcriptional regulatory interactions. *Nucleic Acids Research*.

[23] Zhou Y., Zhou B., Pache L. (2019). Metascape provides a biologist-oriented resource for the analysis of systems-level datasets. *Nature Communications*.

[24] Jafri M. A., Ansari S. A., Alqahtani M. H., Shay J. W. (2016). Roles of telomeres and telomerase in cancer, and advances in telomerase-targeted therapies. *Genome Medicine*.

[25] Shay J. W., Wright W. E. (2010). Telomeres and telomerase in normal and cancer stem cells. *FEBS Letters*.

[26] Ko E., Seo H. W., Jung E. S., Kim B. H., Jung G. (2016). The TERT promoter SNP rs2853669 decreases E2F1 transcription factor binding and increases mortality and recurrence risks in liver cancer. *Oncotarget*.

[27] Tofani S., Barone D., Cintorino M. (2001). Static and ELF magnetic fields induce tumor growth inhibition and apoptosis. *Bioelectromagnetics*.

[28] Zhang L., Wang J., Wang H. (2016). Moderate and strong static magnetic fields directly affect EGFR kinase domain orientation to inhibit cancer cell proliferation. *Oncotarget*.

[29] Papatheofanis F. J. (1990). Use of calcium channel antagonists as magnetoprotective agents. *Radiation Research*.

[30] Li Y., Song L.-Q., Chen M. Q. (2012). Low strength static magnetic field inhibits the proliferation, migration, and adhesion of human vascular smooth muscle cells in a restenosis model through mediating integrins *β*1-FAK, Ca 2+ signaling pathway. *Annals of biomedical engineering.*.

[31] Hung Y. C., Lee J. H., Chen H. M., Huang G. S. (2010). Effects of static magnetic fields on the development and aging of Caenorhabditis elegans. *The Journal of Experimental Biology*.

[32] Lee C. H., Hung Y. C., Huang G. S. (2010). Static magnetic field accelerates aging and development in nematode. *Communicative & Integrative Biology*.

